# Trends in pre-hospital volume resuscitation of blunt trauma patients: a 15-year analysis of the British (TARN) and German (TraumaRegister DGU®) National Registries

**DOI:** 10.1186/s13054-024-04854-x

**Published:** 2024-03-15

**Authors:** M. F. Bath, J. Schloer, J. Strobel, W. Rea, R. Lefering, M. Maegele, H. De’Ath, Z. B. Perkins

**Affiliations:** 1 Centre for Trauma Sciences, Queen Mary, University of London, London, UK; 2 Health Systems Design Group, Department of Engineering, University of Cambridge, Cambridge, UK; 3 Department of Emergency Medicine, Klinikum St. Marien Amberg, Amberg, Germany; 4 London’s Air Ambulance, London, UK; 5 Berufsfeuerwehr Hamburg, Emergency Medical Services, Hamburg, Germany; 6 Institute for Research in Operative Medicine, Cologne Merheim Medical Center, University of Witten/Herdecke, Cologne, Germany

**Keywords:** Trauma, Resuscitation, Crystalloids, Fluids, Outcomes

## Abstract

**Introduction:**

Fluid resuscitation has long been a cornerstone of pre-hospital trauma care, yet its optimal approach remains undetermined. Although a liberal approach to fluid resuscitation has been linked with increased complications, the potential survival benefits of a restrictive approach in blunt trauma patients have not been definitively established. Consequently, equipoise persists regarding the optimal fluid resuscitation strategy in this population.

**Methods:**

We analysed data from the two largest European trauma registries, the UK Trauma Audit and Research Network (TARN) and the German TraumaRegister DGU® (TR-DGU), between 2004 and 2018. All adult blunt trauma patients with an Injury Severity Score > 15 were included. We examined annual trends in pre-hospital fluid resuscitation, admission coagulation function, and mortality rates.

**Results:**

Over the 15-year study period, data from 68,510 patients in the TARN cohort and 82,551 patients in the TR-DGU cohort were analysed. In the TARN cohort, 3.4% patients received pre-hospital crystalloid fluids, with a median volume of 25 ml (20–36 ml) administered. Conversely, in the TR-DGU cohort, 91.1% patients received pre-hospital crystalloid fluids, with a median volume of 756 ml (750–912 ml) administered. Notably, both cohorts demonstrated a consistent year-on-year decrease in the volume of pre-hospital fluid administered, accompanied by improvements in admission coagulation function and reduced mortality rates.

**Conclusion:**

Considerable variability exists in pre-hospital fluid resuscitation strategies for blunt trauma patients. Our data suggest a trend towards reduced pre-hospital fluid administration over time. This trend appears to be associated with improved coagulation function and decreased mortality rates. However, we acknowledge that these outcomes are influenced by multiple factors, including other improvements in pre-hospital care over time. Future research should aim to identify which trauma populations may benefit, be harmed, or remain unaffected by different pre-hospital fluid resuscitation strategies.

**Supplementary Information:**

The online version contains supplementary material available at 10.1186/s13054-024-04854-x.

## Introduction

Fluid resuscitation has been a central component of pre-hospital trauma care for the past 50 years. Despite its wide acceptance as a standard practice, the lack of conclusive evidence demonstrating its benefits and substantial variability in existing guidelines creates a challenge for clinicians [1]. While some guidelines advocate for early and aggressive volume resuscitation with crystalloid fluids, others recommend a more restrictive approach with tolerance of hypotension until bleeding is controlled [1, 2]. There remains an absence of consensus globally in the optimal approach to fluid resuscitation in pre-hospital trauma care [3].

Uncontrolled haemorrhage is the dominant cause for early mortality in patients presenting with severe injury and accounts for the majority of preventable trauma deaths worldwide [4–8]. The majority of these deaths occur soon after injury, often during the pre-hospital phase of care. This critical period before haemorrhage is controlled presents pre-hospital clinicians with a difficult decision; administer fluids, thereby risking aggravating bleeding, or withholding fluids, thereby risking organ ischaemia and hypovolaemic cardiac arrest [9].

Fluid resuscitation strategies have evolved considerably as our understanding of bleeding, shock, and trauma-induced coagulopathy has improved. As early as 1918, practical guidance on the management of traumatic injuries was clear that intravenous fluid administration before haemorrhage control was potentially dangerous [10]. However, by the Vietnam war, aggressive fluid resuscitation with crystalloid fluids had replaced blood transfusion and become the standard of care. This change in practice was largely based on animal haemorrhage experiments performed between 1950s and 60s [11]. While these experiments showed improved survival with fluid administration after controlled blood loss, they were limited in their ability to simulate trauma patients with uncontrolled bleeding and were not able to assess the effects of fluid resuscitation on haemostasis. Eminent academics at the time warned against such aggressive crystalloid resuscitation strategies, pleading for “moderation” and for whole blood to remain the resuscitation fluid of choice for haemorrhage [12].

By the 1990s, animal experiments of uncontrolled haemorrhage provided evidence that aggressive fluid resuscitation before haemorrhage control reduces survival [13–15], with these findings subsequently reflected in a randomised control trial of adult humans with penetrating torso injuries [16]. Furthermore, human observational studies identified a dose-dependent relationship between the volume of resuscitation fluid administered and a host of deleterious complications, including trauma-induced coagulopathy, acute respiratory distress syndrome, multi-organ failure, abdominal compartment syndrome, and extremity compartment syndrome [3, 17, 18]. However, any survival benefit of a restrictive approach has not been shown in patients with blunt trauma [19], and it appears that moderate volumes of pre-hospital resuscitation fluid may be associated with the lowest mortality in this group [20]. Unpicking which patient cohorts are most appropriate for fluid resuscitation and for which a restrictive approach is more suitable remains in debate.

The aim of this study was to describe changes in pre-hospital fluid resuscitation practice over a 15-year period in adult patients with blunt major trauma, by reviewing data from the two largest European trauma registries, the Trauma Audit and Research Network (TARN) in the UK and the TraumaRegister DGU® (TR-DGU) in Germany. The analysis aims to provide insights into the evolving landscape of pre-hospital fluid resuscitation and its potential impact on patient outcomes.

## Methods

### Study design

This was a retrospective review of two large national trauma registries, the UK’s Trauma Audit and Research Network (TARN) registry and the TraumaRegister DGU® of the German Trauma Society (TR-DGU), between 1 January 2004 and 31 December 2018. Ethical approval was provided for this study in the UK by Queen Mary University of London (QMREC2383a) and in Germany by the University of Witten/Herdecke (No. 05/2020).

### Setting

The Emergency Medical Systems in the UK and Germany represent distinct models of pre-hospital care delivery [21]. The UK’s system, an evolution of the Anglo-American ‘scoop and run’ model, prioritises rapid transport of patients to trauma centres with minimal pre-hospital intervention, typically delivered by trained Paramedics and Emergency Medical Technicians [22]. National guidelines for adults with blunt force trauma in the UK recommend a conservative approach to pre-hospital intravenous fluid administration: no fluids if a radial pulse is present, and if absent, fluids are administered in boluses of no more than 250 ml, followed by reassessment and the process repeated until pulse restoration. Furthermore, only personnel trained in advanced life support are authorised to administer intravenous fluids, and this must not delay transport to hospital [2]. In contrast, Germany follows a Franco-German ‘stay and stabilize’ approach, with comprehensive pre-hospital interventions delivered by physicians with an extensive scope of practice. German guidelines recommend volume therapy to maintain stable circulation, endorsing balanced, isotonic crystalloid solutions tailored to the patient’s condition [23].

### Study registries

The TARN registry was founded in 1990 and collects data from 220 Major Trauma Centres (MTC) and trauma units (TU) across the UK [24]. Inclusion criteria are patients who arrive at hospital alive and meet any of the following: death from injury at any point during admission, stay in hospital of longer than three days, need for intensive or high dependency care, or need for inter-hospital transfer for specialist care. The TR-DGU was founded in 1993 and collects data from almost 700 hospitals [25]. While the majority of these hospitals are located in Germany (90%), an increasing number of institutions from other countries are contributing data as well. Inclusion criteria encompass any trauma patient admitted to hospital with subsequent need of Intensive Care Unit (ICU) care, or those who die before admission to ICU. The present study is in line with the publication guidelines of the TARN registry and the TraumaRegister DGU® (TR-DGU project ID 2019-055).

### Data collection

Adult patients (≥ 18 years) who had suffered a blunt mechanism of injury and admitted directly to a major trauma centre with an Injury Severity Score (ISS) > 15 were included in the study. Patients with combined blunt and penetrating injuries, transferred from another hospital to the receiving trauma centre, or with isolated head injuries were excluded. Data collected from each registry included patient age, gender, mechanism of injury, ISS, pre-hospital crystalloid, colloid, and blood product use, initial international normalised ratio (INR) on presentation to hospital, length of hospital stay, and in-hospital mortality.

### Outcomes

The aim of the study was to assess changes and trends in volume resuscitation practice over the past 15 years across both registries. Secondary measures included rates of trauma-induced coagulopathy (defined as an admission INR > 1.2 [26]) and in-hospital mortality rates.

### Statistical analysis

Statistical analyses were performed using GraphPad PRISM v9 (GraphPad, La Jolla, CA). Data distribution assessed visually using histograms. Continuous data are reported as mean ± standard deviation (SD) or as median with interquartile range (IQR), categorical data as frequency (n) and percentage (%) or percentage ± 95% confidence intervals (CI). Cohort characteristics were compared using Welch’s *t* test, *χ*^2^ or Fisher exact tests as appropriate. Trends over time for categorical variables were assessed using *χ*^2^ test for trend. Trends over time for continuous variables were estimated through linear regression models using the slope parameter with 95% CI. Correlation between two variables was assessed using Pearson’s correlation coefficient *r.* Tests were two-sided, and *p* < 0.001 was considered significant.

## Results

### Patient demographics

**Fig. 1:**
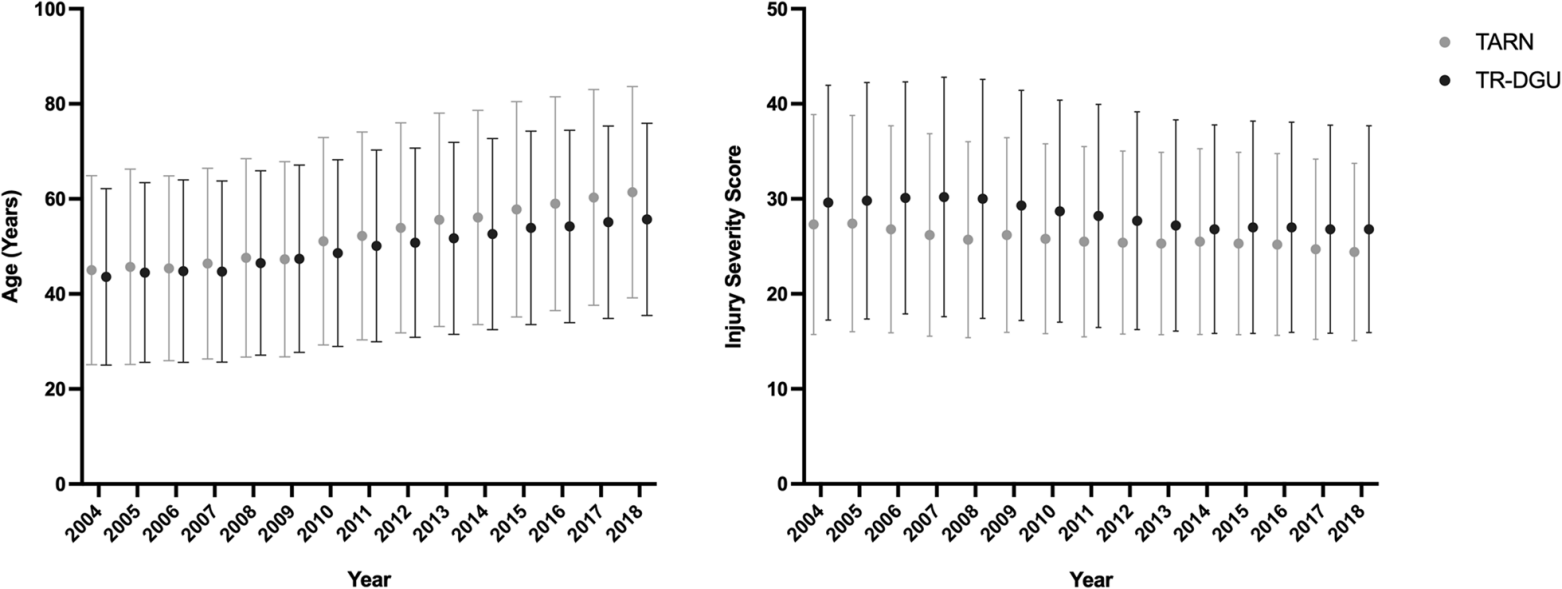
Annual trends in age and injury severity in adult blunt trauma patients. This figure illustrates changes over time in **A** the mean age, and **B** the mean Injury Severity Score (ISS), for adult patients (age ≥ 16) who sustained major blunt force injuries (ISS > 15) and were included in the UK TARN registry or TraumaRegister DGU® between 2004 and 2018. Data is presented mean ± 1 standard Deviation

During the 15-year study period, 68,510 patients were identified from the TARN registry and 82,551 patients from the TR-DGU that met the inclusion criteria for this study. In the TARN cohort, mean age was 56.3 ± 22.7 years, 65.7% were male, and mean ISS was 25.3. On average, patients in the TR-DGU cohort were younger (mean age 51.7 ± 20.2 years), more likely male (71.7%), and more severely injured (mean ISS 27.6) (Table 1). In both cohorts, there was a year-on-year increase in the mean age of patients, while the mean ISS remained relatively consistent throughout the study period (Fig. 1). Road traffic collisions were the most common cause of blunt injury in both registries.

**Table 1:** Baseline characteristics of the study population

	TARN Cohort (*n* = 68,510)	TR-DGU Cohort (*n* = 82,551)	*p*-value
Age, years (range)	56.3 (18–106)	51.7 (18—110)	< 0.001
Male gender	65.7 (65.4–66.1)	71.7 (71.4–72.0)	< 0.001
*Mechanism of injury*			
Road Traffic Collision	41.5 (41.1–41.9)	57.8 (57.5–58.1)	< 0.001
High Energy Fall ^a^	20.3 (20.0–20.6)	19.9 (19.6–20.2)	0.054
Low Energy Fall	29.5 (29.2–29.8)	14.6 (14.4–14.8)	< 0.001
Other	8.7 (8.5–8.9)	7.3 (7.1–7.5)	< 0.001
Injury Severity Score	25.3 ± 9.8	27.6 ± 11.4	< 0.001
*Abbreviated injury scale*			
Head AIS ≥ 2	44.2 (43.8–44.6)	53.5 (53.2–53.8)	< 0.001
Face AIS ≥ 2	18.5 (18.2–18.8)	17.5 (17.2–17.8)	< 0.001
Thorax AIS ≥ 2	58.9 (58.5–59.3)	74.5 (74.2–74.8)	< 0.001
Abdomen AIS ≥ 2	16.1 (15.8–16.4)	25.4 (25.1–25.7)	< 0.001
Pelvis AIS ≥ 2	21.8 (21.5–22.1)	27.2 (26.9–27.5)	< 0.001
Spine AIS ≥ 2	37.2 (36.8–37.6)	40.8 (40.5–41.1)	< 0.001
Extremities AIS ≥ 2	46.0 (45.6–46.4)	59.9 (59.6–60.2)	< 0.001
*Hospital length of stay (days)*			
Survivors	21.5 ± 27.4	23.3 ± 20.8	< 0.001
Deceased	4.9 ± 6.0	6.9 ± 13.1	< 0.001
Mortality	16.7 (16.5–17.0)	15.9 (15.6–16.1)	< 0.001

Data presented as per cent (95% Confidence Interval) or mean ± standard deviation, unless otherwise stated. AIS, Abbreviated Injury Scale

^a^High energy fall defined as ≥ 2 m in TARN cohort and ≥ 3 m in TR-DGU cohort

### Crystalloids

**Fig. 2:**
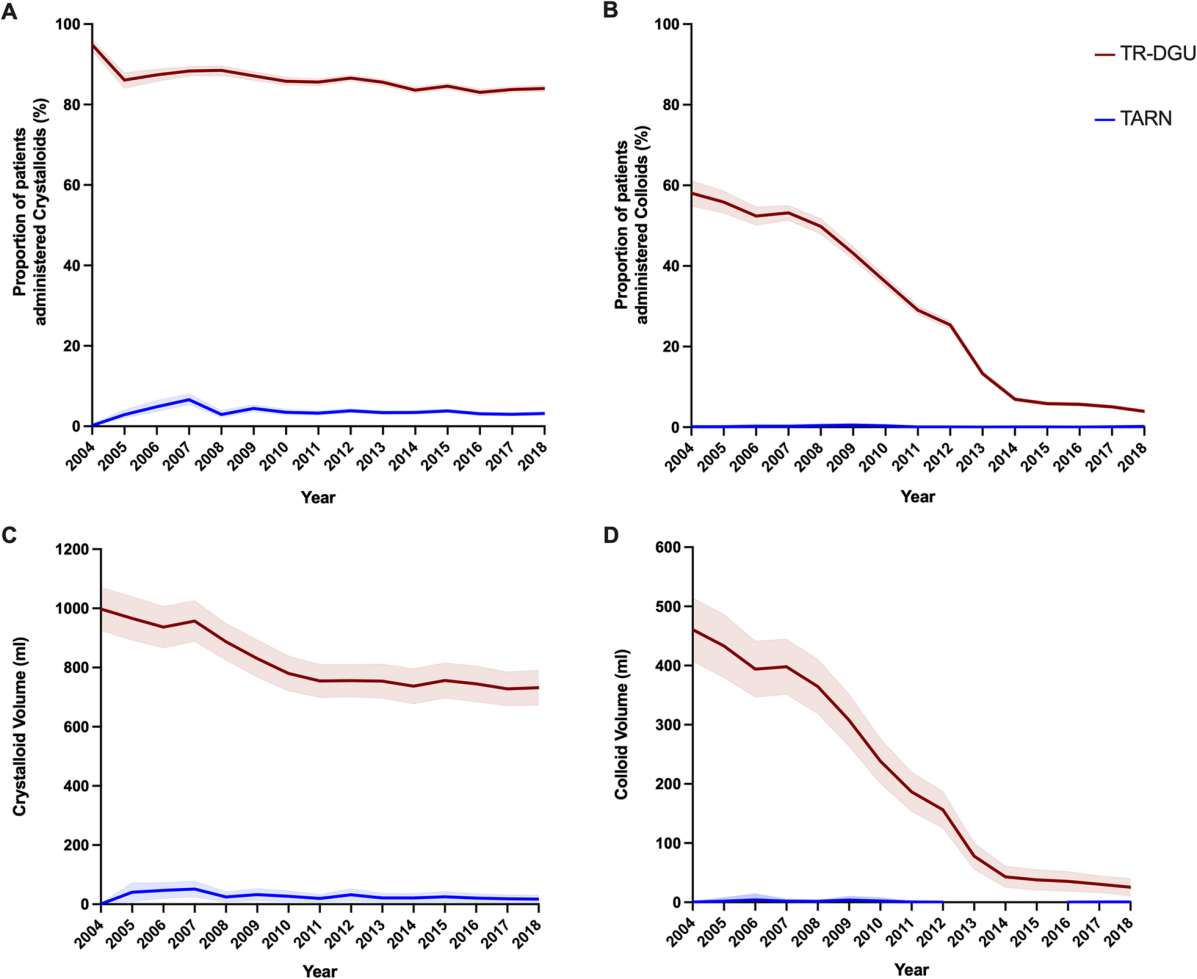
Annual trends in pre-hospital volume resuscitation in 68510 patients included in the UK TARN registry and 82551 patients included in the TraumaRegister DGU® between 2004 and 2018. This figure illustrates the proportion of trauma patients administered **A** crystalloid and **B** colloid resuscitation fluids, data presented as percent with 95% Confidence Interval, and the average volume of **C** crystalloid and **D** colloid resuscitation fluids administered, data presented as mean ± 1 standard deviation

Overall, 2,336 patients (3.4% [95% CI 3.3–3.5]) in the TARN cohort were administered crystalloid fluids during the pre-hospital phase of care. The proportion of TARN patients administered crystalloid fluids remained similar each year during the study period (*p* = 0.054, Fig. 2A). By comparison, 70,230 patients (91.1% [95% CI 90.9–91.3]) in the TR-DGU cohort were administered crystalloid fluids during the pre-hospital phase of care. There was a small but significant decrease in the proportion of TR-DGU patients administered crystalloid fluids over the study period, from 93.2% in 2004 to 89.7% in 2018 (*p* < 0.0001, Fig. 2A).

The median volume of crystalloid fluid administered to TARN patients was significantly less than the volume administered to TR-DGU patients (TARN 25mls (IQR 20–32) versus TR-DGU 756mls (IQR 750–912), *p* < 0.001). In both cohorts, there was a year-on-year decrease in the volume of crystalloid administered to patients (TARN: slope − 1.12 (95% CI − 1.14 to − 1.11), *p* < 0.001; TR-DGU: slope − 15.4 (95% CI − 15.6 to − 15.3), *p* < 0.001, Fig. 2C).

### Colloids

Overall, 83 patients (0.1% [95% CI 0.1–0.2]) in the TARN cohort were administered colloids during the pre-hospital phase of care. There was a small reduction in the proportion of TARN patients administered colloid each year (*p* = 0.01, Fig. 2B). By comparison, 15,410 patients (18.7% [95% CI 18.4–18.9]) in the TR-DGU cohort were administered colloids during the pre-hospital phase of care. There was a significant decrease in the proportion of TR-DGU patients administered colloid each year over the study period, from 58.1% in 2004 to 3.9% in 2018 (*p* < 0.001, Fig. 2B).

The median volume of colloid fluid administered to TARN patients was significantly lower than the volume administered to TR-DGU patients (TARN: 0 ml (IQR 0–0 ml) versus TR-DGU 186 ml (IQR 40–379 ml), *p* < 0.001). In both cohorts, there was a year-on-year decrease in the volume of colloid administered to patients (TARN: slope − 0.15 (–0.15 to − 0.14), *p* < 0.001; TR-DGU: slope − 34.0 (− 34.0 to − 33.9), *p* < 0.001, Fig. 2D).

### Blood products

Blood product transfusions were administered to patients in the TARN cohort during the pre-hospital setting. Overall, 134 patients (0.2%) were transfused packed red blood cells, and 95 patients (0.1%) were transfused fresh frozen plasma. The majority of transfusions (216/229, 94%) were administered after 2012. No patients in the TR-DGU cohort were transfused blood products during the pre-hospital phase of care during the study period.

### Rates of trauma-induced coagulopathy

**Fig. 3:**
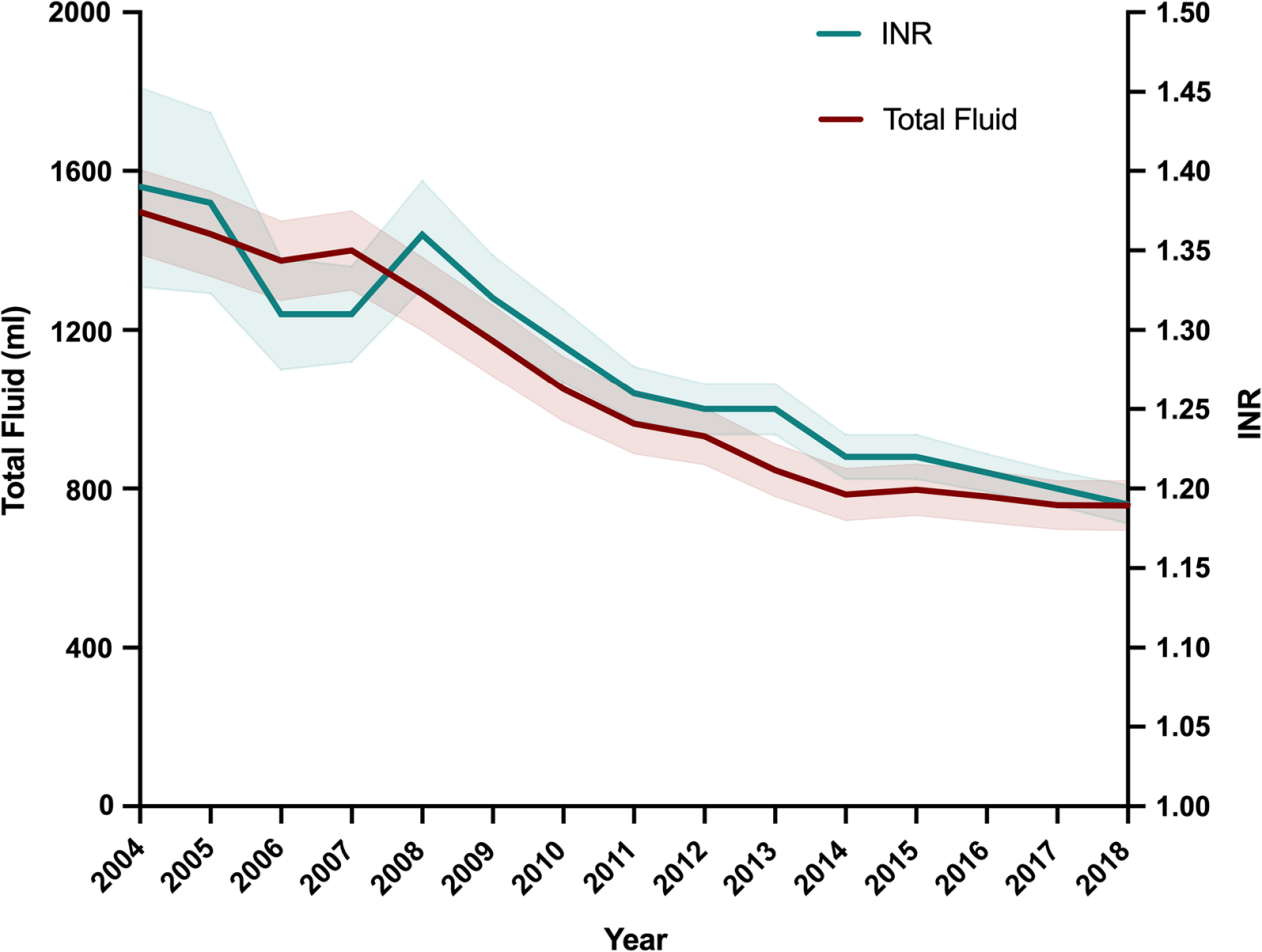
Relationship between the volume of resuscitation fluid administered and admission coagulation function (International Normalised Ratio, INR) in 76,935 patients included in the TraumaRegister DGU®. Fluid volume data are presented as mean ± 1 standard deviation and INR data as mean with 95% confidence interval

Coagulation data were only available in the TR-DGU cohort. An admission INR result was recorded in 93.2% (76,935 out of 82,551) of TR-DGU cases. The mean INR during the study period was 1.24 ± 0.66 with a significant year-on-year improvement in admission coagulation function (INR) over the study period (slope − 0.0132 (95% CI − 0.0133 to − 0.0130; *p* < 0.0001). There was a significant positive correlation between the reduction in pre-hospital fluid administration and improvement in admission coagulation function over the study period (*r* = 0.947 (95% CI 0.845–0.983), *p* < 0.0001, Fig. 3).

### Mortality

**Fig. 4:**
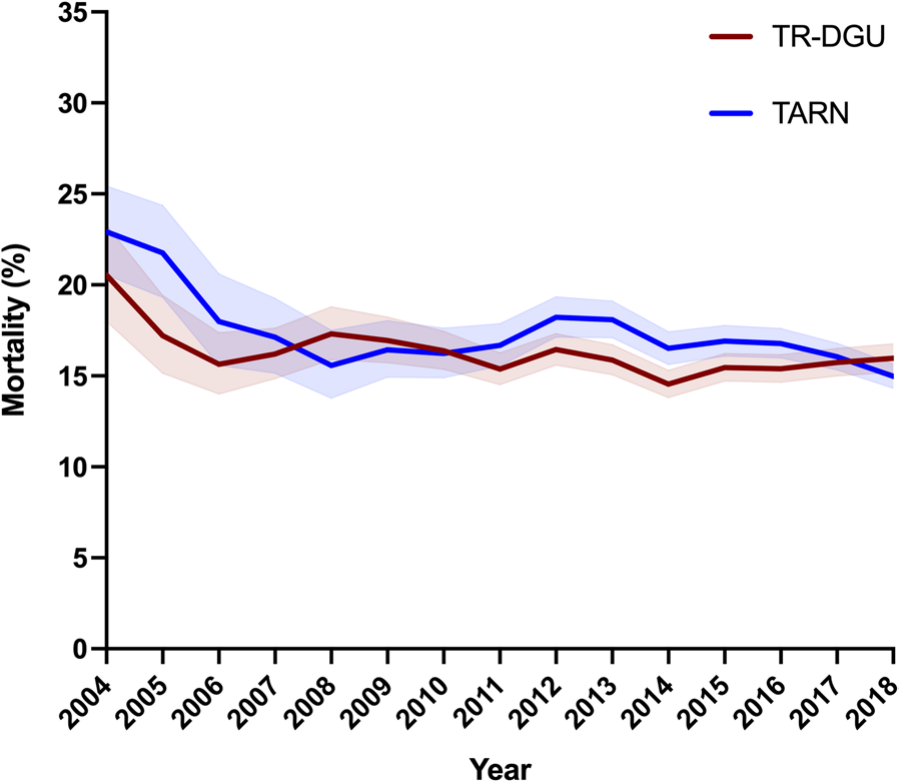
Mortality rate following blunt trauma in 68,510 patients included in the UK TARN registry and 82,551 patients included in the TraumaRegister DGU® between 2004 and 2018. Data presented as mortality rate (%) with 95% confidence intervals

The overall mortality was 16.7% (95% CI 16.5–17.0) in the TARN cohort and 15.9% (95% CI 15.6–16.1) in the TR-DGU cohort. In both the TARN and TR-DGU registries, there was a small but significant year-on-year decrease in mortality (*p* < 0.001 and *p* < 0.001, respectively, Fig. 4). The decrease in mortality over the study period was similar in both groups (TARN: slope –0.003 (95% CI − 0.005 to − 0.001) versus TR-DGU: slope − 0.002 (95% CI − 0.003 to − 0.001); *p* = 0.338).

## Discussion

This study compared trends in pre-hospital fluid administration following blunt trauma in the UK (TARN) and Germany (TR-DGU) between 2004 and 2018. We observed considerable variability in pre-hospital fluid resuscitation practice between countries, with the UK adopting a restrictive approach and Germany favouring a more liberal approach. Notably, both cohorts demonstrated a consistent year-on-year decrease in the volume of pre-hospital fluid administered, which corresponded with modest reductions in trauma-induced coagulopathy and mortality rates.

The liberal use of crystalloid fluids in trauma patients has been a standard practice since the Vietnam war [27]. In Germany, this approach remains integral to current national trauma guidelines [23], while in the UK, there has been a shift towards a more restrictive approach in recent years [2]. Our study observed a striking contrast in national practice, with TR-DGU patients receiving nearly 20 times more crystalloid fluid compared to TARN patients. One of the factors contributing to the adoption of a restrictive fluid strategy in UK practice has been the growing recognition of the impact of crystalloid fluids on coagulation dysfunction in trauma patients and the adverse consequences of this complication in bleeding trauma patients [28]. Interestingly, despite the substantial differences in volume resuscitation strategies, we observed similar trauma mortality rates in both countries (with an almost parallel reduction in year-on-year mortality rates). This suggests that pre-hospital fluid resuscitation strategies may play a lesser role in the overall mortality of major trauma patients compared to other aspects of care, highlighting the importance of identifying the patient cohorts that may benefit from this intervention.

Blunt trauma patients with pre-hospital hypotension constitute a subgroup of particular clinical importance. Resuscitation guidelines in both the UK and Germany recommend volume resuscitation in these patients, an approach supported by recent studies [2, 23]. Secondary analysis of the PROMMTT Study suggests that moderate pre-hospital crystalloid administration (median 700 ml) correlates with reduced mortality compared to administering no fluids [29]. Similarly, pilot randomised trial data and secondary analysis of the PAMPer trial data demonstrated that resuscitation with moderate volumes (250–1250 ml) of pre-hospital crystalloids was associated with improved 24-h mortality in hypotensive blunt trauma patients, compared with lower or higher volumes [20, 30]. While these studies provide valuable insights into volume resuscitation strategies for shocked patients following blunt injury, the scope of our study is broader, evaluating fluid resuscitation practice in both shocked and non-shocked patients, and longer-term outcomes across the UK and Germany. Our data does not isolate the subgroup of shocked patients for direct comparison, further emphasising the need for future research to delineate the precise role of fluid resuscitation in different trauma subgroups.

Crystalloids have known negative effects on coagulation function in trauma patients, including dilutional effects on the coagulation factors [31], increased fibrinolysis [32], and glycocalyceal shedding [33]. A sub-analysis of a large multi-centre randomised trial demonstrated that pre-hospital crystalloid administration in trauma patients is associated with increased rates of coagulopathy [34], as well as an elevated risk of acute respiratory distress syndrome [34], corroborating findings from earlier registry-based studies [35]. Our study findings were consistent with these observations, as we observed a strong correlation between the volume of pre-hospital fluid administered and admission coagulation function. Both the TARN and TR-DGU registries demonstrated a significant reduction in crystalloid use over the study period. Similar reductions have been reported in other countries, most notably a recent study from the US Department of Defence Trauma Registry, which showed a decrease in the number of injured patients receiving pre-hospital crystalloids between 2007 and 2020 [19].

Reassuringly, the overall use of colloids for fluid resuscitation in both cohorts was low. Crystalloids over colloids as the choice of resuscitation fluid has been recommended by both UK and German groups for several years. Indeed, colloids are now known to be harmful in their use compared to crystalloids, conferring no survival benefit and increasing morbidity rates and adverse events [36, 37], and certain colloids are now no longer available in pre-hospital care in either country. Use of colloids in the TARN group remained low throughout the study period, and while its initial use was higher in the TR-DGU group (with over half of patients receiving colloids in 2004), its use significantly dropped during the study period, reaching levels comparable to the TARN registry.

Early transfusion of blood products in bleeding trauma patients has been associated with improved patient outcomes [38]. Our data reflect this recent advancement in understanding, with a small but increasing number of TARN patients receiving pre-hospital blood products rather than crystalloids from 2012 onwards. Recently, pre-hospital blood transfusion has been implemented in Germany, with the first transfusion of pre-hospital blood products occurring in 2019 [39]; however, its availability remains limited to select rescue service locations [40]. Although not explored in our study, there remains ongoing equipoise regarding the optimal strategy for blood product transfusion in trauma [41]. A recent multi-centre randomised controlled trial did not demonstrate an overall mortality benefit in the use of packed red blood cells and plasma in shocked trauma patients compared to crystalloid fluids alone [42].

It is important to acknowledge the limitations of our study, which primarily stem from its observational nature. As such, we can only describe correlations and not demonstrate causative associations based on our results. Furthermore, being a retrospective database analysis, the study is susceptible to inherent recall bias and relies on the accuracy of the data input, with potential confounding factors left unaccounted. Variables in the TARN registry have changed over time, meaning the accuracy of certain measures may be variable across the study period. Additionally, complete assessment of patient coagulopathy rates was limited both by the availability of coagulation data solely in the TR-DGU cohort and in INR being the sole measure of coagulopathy. The inclusion criteria for the respective registries slightly differ, resulting in slight differences between the two cohorts (e.g. TARN registry has a higher proportion of elderly falls from standing, while the TR-DGU registry has a higher proportion of younger patents in high energy trauma), precluding any direct comparisons between the two groups being made, and timing of patient presentation following injury or type of transportation to hospital has not been accounted for. However, the strengths of this study lie in the inclusion of a large patient population over an extended study period, allowing for important insights into current trends in pre-hospital fluid resuscitation practice.

In conclusion, this study reveals considerable differences in current pre-hospital fluid resuscitation practice in blunt trauma patients, with the UK favouring a more restrictive approach and Germany a more liberal approach. We also observed a close correlation between reduced pre-hospital crystalloid use and lower admission coagulopathy rates. Despite the substantial differences in resuscitation practice, unadjusted mortality rates between the two groups were largely similar. This suggests that the impact of pre-hospital fluid resuscitation on different trauma patient subgroups is still not well-understood. Future research should aim to understand which trauma populations may benefit, be harmed, or remain unaffected by different pre-hospital fluid resuscitation strategies, thus facilitating the development of more tailored approaches to optimise patient outcomes.

### Supplementary Information

**Additional file 1**. Study data.

## Data Availability

All data relevant to the study are included in the article or uploaded as Additional file 1. All data presented in this investigation were provided by the UK Trauma Audit and Research Network (TARN) and the TraumaRegister DGU (TR-DGU)® of the German Akademie der Unfallchirurgie GmbH (AUC). The original data used in this study are available from TARN (www.tarn.ac.uk) and the AUC (support-tr@auc-online.de) on reasonable request.
