# Supplementary material for: Trends in pre-hospital volume resuscitation of blunt trauma patients: a 15-year analysis of the British (TARN) and German (TraumaRegister DGU®) National Registries
Source: Crit Care. 2024 Mar 15;28:81. doi: 10.1186/s13054-024-04854-x (PMC10941386; doi:10.1186/s13054-024-04854-x)
Supplement: Supplementary file 1 — Additional file 1. Study data. [file 13054_2024_4854_MOESM1_ESM.docx]

| Characteristic | Age | | | | | |
| --- | --- | --- | --- | --- | --- | --- |
| Cohort | TR-DGU | | | TARN | | |
| Measures | Cases per year | Mean | SD | Cases per year | Mean | SD |
| 2004 | 930 | 43.6 | 18.57 | 1179 | 45 | 19.88 |
| 2005 | 1244 | 44.5 | 18.91 | 1065 | 45.7 | 20.56 |
| 2006 | 1829 | 44.8 | 19.18 | 946 | 45.4 | 19.46 |
| 2007 | 2759 | 44.7 | 19.05 | 1347 | 46.4 | 20.07 |
| 2008 | 2675 | 46.5 | 19.4 | 1494 | 47.6 | 20.86 |
| 2009 | 3540 | 47.4 | 19.72 | 2261 | 47.3 | 20.52 |
| 2010 | 4864 | 48.6 | 19.65 | 2899 | 51.1 | 21.84 |
| 2011 | 6662 | 50.1 | 20.17 | 4017 | 52.2 | 21.88 |
| 2012 | 7188 | 50.8 | 19.91 | 4723 | 53.9 | 22.11 |
| 2013 | 7514 | 51.7 | 20.21 | 5657 | 55.6 | 22.47 |
| 2014 | 8444 | 52.6 | 20.11 | 6685 | 56.1 | 22.55 |
| 2015 | 8654 | 53.9 | 20.35 | 7535 | 57.8 | 22.66 |
| 2016 | 8840 | 54.2 | 20.25 | 8427 | 59 | 22.51 |
| 2017 | 9003 | 55.1 | 20.25 | 9615 | 60.3 | 22.7 |
| 2018 | 8405 | 55.7 | 20.21 | 10861 | 61.4 | 22.22 |
| Total | 82551 | 51.7 | 20.89 | 68711 | 56.3 | 22.65 |

| Characteristic | Injury Severity Score | | | | | |
| --- | --- | --- | --- | --- | --- | --- |
| Cohort | TR-DGU | | | TARN | | |
| Measures | Cases per year | Mean | SD | Cases per year | Mean | SD |
| 2004 | 930 | 29.6 | 12.36 | 1179 | 27.3 | 11.57 |
| 2005 | 1244 | 29.8 | 12.45 | 1065 | 27.4 | 11.39 |
| 2006 | 1829 | 30.1 | 12.22 | 946 | 26.8 | 10.89 |
| 2007 | 2759 | 30.2 | 12.61 | 1347 | 26.2 | 10.66 |
| 2008 | 2675 | 30 | 12.58 | 1494 | 25.7 | 10.31 |
| 2009 | 3540 | 29.3 | 12.11 | 2261 | 26.2 | 10.25 |
| 2010 | 4864 | 28.7 | 11.68 | 2899 | 25.8 | 9.99 |
| 2011 | 6662 | 28.2 | 11.74 | 4017 | 25.5 | 10.01 |
| 2012 | 7188 | 27.7 | 11.46 | 4723 | 25.4 | 9.64 |
| 2013 | 7514 | 27.2 | 11.12 | 5657 | 25.3 | 9.6 |
| 2014 | 8444 | 26.8 | 10.97 | 6685 | 25.5 | 9.78 |
| 2015 | 8654 | 27 | 11.17 | 7535 | 25.3 | 9.6 |
| 2016 | 8840 | 27 | 11.06 | 8427 | 25.2 | 9.56 |
| 2017 | 9003 | 26.8 | 10.95 | 9615 | 24.7 | 9.48 |
| 2018 | 8405 | 26.8 | 10.88 | 10861 | 24.4 | 9.33 |
| Total | 82551 | 27.6 | 11.43 | 68711 | 25.3 | 9.76 |

| Characteristic | Pre-hospital Crystalloid (n,%) | | | | | |
| --- | --- | --- | --- | --- | --- | --- |
| Cohort | TR-DGU | | | TARN | | |
| Measures | Cases per year | Number | Percentage | Cases per year | Number | Percentage |
| 2004 | 882 | 822 | 0.932 | 1179 | 2 | 0.00169635 |
| 2005 | 1144 | 1071 | 0.936 | 1065 | 31 | 0.02910798 |
| 2006 | 1712 | 1598 | 0.933 | 946 | 46 | 0.04862579 |
| 2007 | 2597 | 2437 | 0.938 | 1347 | 89 | 0.06607275 |
| 2008 | 2507 | 2367 | 0.944 | 1494 | 44 | 0.02945114 |
| 2009 | 3317 | 3085 | 0.93 | 2261 | 100 | 0.04422822 |
| 2010 | 4510 | 4173 | 0.925 | 2899 | 101 | 0.0348396 |
| 2011 | 6218 | 5702 | 0.917 | 4017 | 131 | 0.0326114 |
| 2012 | 6726 | 6224 | 0.925 | 4723 | 183 | 0.03874656 |
| 2013 | 7049 | 6427 | 0.912 | 5657 | 192 | 0.03394025 |
| 2014 | 7796 | 7059 | 0.905 | 6685 | 231 | 0.03455497 |
| 2015 | 8099 | 7320 | 0.904 | 7535 | 289 | 0.03835435 |
| 2016 | 8242 | 7342 | 0.891 | 8427 | 263 | 0.03120921 |
| 2017 | 8423 | 7541 | 0.895 | 9615 | 288 | 0.0299532 |
| 2018 | 7870 | 7062 | 0.897 | 10861 | 346 | 0.0318571 |
| Total | 77092 | 70230 | 0.911 | 68711 | 2336 | 0.03399747 |

| Characteristic | Pre-hospital Crystalloid (ml) | | | | | |
| --- | --- | --- | --- | --- | --- | --- |
| Cohort | TR-DGU | | | TARN | | |
| Measures | Cases per year | Mean | SD | Cases per year | Mean | SD |
| 2004 | 930 | 998 | 73.503 | 1179 | 0.85 | 2.0585 |
| 2005 | 1244 | 965.9 | 73.787 | 1065 | 40.35 | 32.229 |
| 2006 | 1829 | 936.6 | 70.606 | 946 | 47.01 | 27.0806 |
| 2007 | 2759 | 957 | 69.179 | 1347 | 51.15 | 26.7119 |
| 2008 | 2675 | 887.5 | 62.396 | 1494 | 24.7 | 18.3191 |
| 2009 | 3540 | 830.6 | 63.269 | 2261 | 32.55 | 19.9705 |
| 2010 | 4864 | 780.1 | 59.371 | 2899 | 26.91 | 18.7931 |
| 2011 | 6662 | 754.7 | 56.468 | 4017 | 19.71 | 13.5846 |
| 2012 | 7188 | 755.5 | 55.436 | 4723 | 32.05 | 20.3737 |
| 2013 | 7514 | 754 | 57.883 | 5657 | 21.51 | 15.9907 |
| 2014 | 8444 | 736.9 | 59.869 | 6685 | 21.03 | 15.5259 |
| 2015 | 8654 | 756.3 | 59.579 | 7535 | 25.49 | 18.2341 |
| 2016 | 8840 | 745 | 60.783 | 8427 | 20.97 | 15.2103 |
| 2017 | 9003 | 728 | 57.601 | 9615 | 18.33 | 13.6286 |
| 2018 | 8405 | 731.8 | 59.855 | 10861 | 17.39 | 13.1671 |
| Total | 82551 | 772.5 | 60.42 | 68711 | 22.89 | 16.6769 |

| Characteristic | Pre-hospital Colloid (n,%) | | | | | |
| --- | --- | --- | --- | --- | --- | --- |
| Cohort | TR-DGU | | | TARN | | |
| Measures | Cases per year | Number | Percentage | Cases per year | Number | Percentage |
| 2004 | 930 | 540 | 0.581 | 1179 | 1 | 0.00084818 |
| 2005 | 1244 | 695 | 0.559 | 1065 | 1 | 0.00093897 |
| 2006 | 1829 | 958 | 0.524 | 946 | 2 | 0.00211416 |
| 2007 | 2759 | 1467 | 0.532 | 1347 | 3 | 0.00222717 |
| 2008 | 2675 | 1333 | 0.498 | 1494 | 6 | 0.00401606 |
| 2009 | 3540 | 1528 | 0.432 | 2261 | 12 | 0.00530739 |
| 2010 | 4864 | 1753 | 0.36 | 2899 | 10 | 0.00344947 |
| 2011 | 6662 | 1935 | 0.29 | 4017 | 2 | 0.00049788 |
| 2012 | 7188 | 1824 | 0.254 | 4723 | 3 | 0.00063519 |
| 2013 | 7514 | 999 | 0.133 | 5657 | 2 | 0.00035354 |
| 2014 | 8444 | 584 | 0.069 | 6685 | 3 | 0.00044877 |
| 2015 | 8654 | 506 | 0.058 | 7535 | 4 | 0.00053086 |
| 2016 | 8840 | 502 | 0.057 | 8427 | 3 | 0.000356 |
| 2017 | 9003 | 455 | 0.051 | 9615 | 11 | 0.00114405 |
| 2018 | 8405 | 331 | 0.039 | 10861 | 20 | 0.00184145 |
| Total | 82551 | 15410 | 0.187 | 68711 | 83 | 0.00120796 |

| Characteristic | Pre-hospital Colloid (ml) | | | | | |
| --- | --- | --- | --- | --- | --- | --- |
| Cohort | TR-DGU | | | TARN | | |
| Measures | Cases per year | Mean | SD | Cases per year | Mean | SD |
| 2004 | 930 | 460.6 | 53.601 | 1179 | 0.42 | 1.4562 |
| 2005 | 1244 | 433.1 | 53.461 | 1065 | 1.88 | 6.1285 |
| 2006 | 1829 | 394 | 47.279 | 946 | 4.23 | 10.2782 |
| 2007 | 2759 | 398.1 | 47.007 | 1347 | 1.86 | 4.0843 |
| 2008 | 2675 | 364.5 | 45.88 | 1494 | 1.54 | 2.6475 |
| 2009 | 3540 | 307.9 | 44.366 | 2261 | 3.89 | 6.4735 |
| 2010 | 4864 | 238.5 | 38.077 | 2899 | 2.2 | 5.7511 |
| 2011 | 6662 | 186.3 | 33.45 | 4017 | 0.37 | 1.7638 |
| 2012 | 7188 | 156.3 | 31.27 | 4723 | 0.25 | 1.0691 |
| 2013 | 7514 | 77.8 | 22.276 | 5657 | 0.04 | 0.2397 |
| 2014 | 8444 | 43.1 | 18.028 | 6685 | 0.19 | 1.2532 |
| 2015 | 8654 | 37.8 | 17.239 | 7535 | 0.09 | 0.4031 |
| 2016 | 8840 | 35.3 | 16.61 | 8427 | 0.19 | 1.1879 |
| 2017 | 9003 | 30.4 | 14.383 | 9615 | 0.37 | 1.5334 |
| 2018 | 8405 | 25.4 | 14.668 | 10861 | 0.54 | 1.6473 |
| Total | 82551 | 126.62 | 30.964 | 68711 | 0.62 | 2.5881 |

| Characteristic | Pre-hospital Red Blood Cells (n,%) | | | | | |
| --- | --- | --- | --- | --- | --- | --- |
| Cohort | TR-DGU | | | TARN | | |
| Measures | Cases per year | Number | Percentage | Cases per year | Number | Percentage |
| 2004 | 930 | 0 | 0 | 1179 | 0 | 0 |
| 2005 | 1244 | 0 | 0 | 1065 | 0 | 0 |
| 2006 | 1829 | 0 | 0 | 946 | 0 | 0 |
| 2007 | 2759 | 0 | 0 | 1345 | 2 | 0.001 |
| 2008 | 2675 | 0 | 0 | 1494 | 0 | 0 |
| 2009 | 3540 | 0 | 0 | 2260 | 1 | 0 |
| 2010 | 4864 | 0 | 0 | 2898 | 1 | 0 |
| 2011 | 6662 | 0 | 0 | 4013 | 4 | 0.1 |
| 2012 | 7188 | 0 | 0 | 4721 | 2 | 0 |
| 2013 | 7514 | 0 | 0 | 5651 | 6 | 0.1 |
| 2014 | 8444 | 0 | 0 | 6678 | 7 | 0.1 |
| 2015 | 8654 | 0 | 0 | 7516 | 19 | 0.3 |
| 2016 | 8840 | 0 | 0 | 8387 | 40 | 0.5 |
| 2017 | 9003 | 0 | 0 | 9584 | 31 | 0.3 |
| 2018 | 8405 | 0 | 0 | 10840 | 21 | 0.2 |
| Total | 82551 | 0 | 0 | 68577 | 134 | 0.2 |

| Characteristic | Pre-hospital Fresh Frozen Plasma (n,%) | | | | | |
| --- | --- | --- | --- | --- | --- | --- |
| Cohort | TR-DGU | | | TARN | | |
| Measures | Cases per year | Number | Percentage | Cases per year | Number | Percentage |
| 2004 | 930 | 0 | 0 | 1179 | 0 | 0 |
| 2005 | 1244 | 0 | 0 | 1065 | 0 | 0 |
| 2006 | 1829 | 0 | 0 | 946 | 0 | 0 |
| 2007 | 2759 | 0 | 0 | 1347 | 0 | 0 |
| 2008 | 2675 | 0 | 0 | 1494 | 0 | 0 |
| 2009 | 3540 | 0 | 0 | 2260 | 1 | 0 |
| 2010 | 4864 | 0 | 0 | 2897 | 2 | 0.1 |
| 2011 | 6662 | 0 | 0 | 4017 | 0 | 0 |
| 2012 | 7188 | 0 | 0 | 4723 | 0 | 0 |
| 2013 | 7514 | 0 | 0 | 5657 | 0 | 0 |
| 2014 | 8444 | 0 | 0 | 6682 | 3 | 0 |
| 2015 | 8654 | 0 | 0 | 7530 | 5 | 0.1 |
| 2016 | 8840 | 0 | 0 | 8416 | 11 | 0.1 |
| 2017 | 9003 | 0 | 0 | 9582 | 33 | 0.3 |
| 2018 | 8405 | 0 | 0 | 10821 | 40 | 0.4 |
| Total | 82551 | 0 | 0 | 68616 | 95 | 0.1 |

| Characteristic | Admission International Normalised Ratio (INR) | | | | | |
| --- | --- | --- | --- | --- | --- | --- |
| Cohort | TR-DGU | | | TARN | | |
| Measures | Cases per year | Mean | SD | Cases per year | Mean | SD |
| 2004 | 778 | 1.39 | 0.9 | 1179 | na | na |
| 2005 | 1030 | 1.38 | 0.93 | 1065 | na | na |
| 2006 | 1646 | 1.31 | 0.73 | 946 | na | na |
| 2007 | 2511 | 1.31 | 0.76 | 1347 | na | na |
| 2008 | 2409 | 1.36 | 0.85 | 1494 | na | na |
| 2009 | 3155 | 1.32 | 0.76 | 2260 | na | na |
| 2010 | 4308 | 1.29 | 0.78 | 2897 | na | na |
| 2011 | 6142 | 1.26 | 0.68 | 4017 | na | na |
| 2012 | 6721 | 1.25 | 0.67 | 4723 | na | na |
| 2013 | 7097 | 1.25 | 0.7 | 5657 | na | na |
| 2014 | 8005 | 1.22 | 0.63 | 6682 | na | na |
| 2015 | 8192 | 1.22 | 0.64 | 7530 | na | na |
| 2016 | 8393 | 1.21 | 0.57 | 8416 | na | na |
| 2017 | 8565 | 1.2 | 0.54 | 9582 | na | na |
| 2018 | 8026 | 1.19 | 0.56 | 10821 | na | na |
| Total | 76978 | 1.24 | 0.66 | 68616 | na | na |

| Characteristic | Mortality (n,%) | | | | | |
| --- | --- | --- | --- | --- | --- | --- |
| Cohort | TR-DGU | | | TARN | | |
| Measures | Cases per year | Number | Percentage | Cases per year | Number | Percentage |
| 2004 | 930 | 191 | 0.205 | 1179 | 269 | 0.229 |
| 2005 | 1244 | 214 | 0.172 | 1065 | 230 | 0.218 |
| 2006 | 1829 | 286 | 0.156 | 946 | 168 | 0.18 |
| 2007 | 2759 | 447 | 0.162 | 1347 | 229 | 0.171 |
| 2008 | 2675 | 463 | 0.173 | 1494 | 232 | 0.156 |
| 2009 | 3540 | 600 | 0.169 | 2261 | 370 | 0.164 |
| 2010 | 4864 | 797 | 0.164 | 2899 | 468 | 0.162 |
| 2011 | 6662 | 1024 | 0.154 | 4017 | 668 | 0.167 |
| 2012 | 7188 | 1182 | 0.164 | 4723 | 858 | 0.182 |
| 2013 | 7514 | 1193 | 0.259 | 5657 | 1021 | 0.181 |
| 2014 | 8444 | 1228 | 0.145 | 6685 | 1102 | 0.165 |
| 2015 | 8654 | 1338 | 0.155 | 7535 | 1272 | 0.169 |
| 2016 | 8840 | 1361 | 0.154 | 8427 | 1411 | 0.168 |
| 2017 | 9003 | 1417 | 0.157 | 9615 | 1540 | 0.16 |
| 2018 | 8405 | 1342 | 0.16 | 10861 | 1622 | 0.15 |
| Total | 82551 | 13083 | 0.158 | 68711 | 11460 | 0.167 |
